# Intracolonic Treatment with a Rifamycin SV In Situ Gelling Formulation Ameliorates Macroscopic and Histological Damage in an Acute Rat Model of Oxazolone-Induced Colitis: A Proof-of-Concept Study

**DOI:** 10.3390/jcm15166409

**Published:** 2026-08-19

**Authors:** Katia Mangano, Gian Marco Leone, Roberto Di Marco, Caterina Aiello, Cinzia Quattrocchi, Luigi Longo, Stefania Pagani, Mara Gerloni

**Affiliations:** 1 Department of Biomedical and Biotechnological Sciences, University of Catania, 95125 Catania, Italy; katia.mangano@unict.it (K.M.); g.marco-94@outlook.it (G.M.L.); 2 Department of Drug and Health Sciences, University of Catania, 95125 Catania, Italy; roberto.dimarco@unict.it; 3 Cosmo SpA, Via C. Colombo 1, 20045 Lainate, Italy; labct@cosmohc.com (C.A.); cinzia.quattrocchi@gmail.com (C.Q.); spagani@cosmohc.com (S.P.); 4 Cosmo N.V., 7th Floor 91/94 North Wall Quay, D01 H7V7 Dublin, Ireland; llongo@cosmohc.com; 5 Cosmobiolabs, 6199 Cornerstone Court E., Suite 101, San Diego, CA 92121, USA

**Keywords:** ulcerative colitis, oxazolone, rat model, preclinical study, CB-01-35, inflammation

## Abstract

**Background/Objectives**: Ulcerative colitis (UC) is a chronic inflammatory bowel disease characterized by persistent inflammation of the colonic mucosa, resulting from a complex interplay between epithelial barrier dysfunction, dysregulated immune responses, and alterations in the gut microbiota. Despite advances in therapeutic strategies, current treatments for UC remain suboptimal. Many conventional and biologic therapies are associated with systemic side effects due to non-specific distribution, which can limit their long-term use. Novel acting therapies are needed for ulcerative colitis. This study evaluated the efficacy of intracolonic Rifamycin SV in situ gelling formulation (CB-01-35) in a rat model of oxazolone-induced colitis. **Methods**: Acute colitis was induced in female Wistar rats using oxazolone. Animals were treated for three days with CB-01-35 (80 mg/kg, intracolonic), Vehicle, Asacol, or dexamethasone. Clinical parameters, colon weight, macroscopic damage score (MDS), mucosal damage area, and histological score were assessed. **Results**: CB-01-35 significantly reduced MDS compared with Vehicle (*p* < 0.05) and showed a trend toward reduced mucosal damage area (*p* = 0.06). Histological analysis confirmed significant improvement, with reduced leukocyte infiltration and epithelial damage. No significant effects were observed on body weight or colon weight. Comparator treatments showed limited or inconsistent efficacy. **Conclusions**: CB-01-35 demonstrated therapeutic activity in this preclinical model, supporting further investigation as a locally acting treatment for colitis. These findings represent an initial proof-of-concept and warrant confirmation in larger and mechanistic studies.

## 1. Introduction

Ulcerative colitis (UC) is a chronic inflammatory bowel disease characterized by relapsing inflammation of the colonic mucosa, driven by a complex interplay between epithelial barrier dysfunction, immune dysregulation, and alterations in the gut microbiota [1,2,3]. Despite the availability of multiple therapeutic options, including aminosalicylates, corticosteroids, immunosuppressants, and biologics, a substantial proportion of patients fail to achieve sustained remission or develop treatment-related adverse effects [4,5]. Distal ulcerative colitis (UC), and in particular ulcerative proctitis, remains a clinically significant and under-served condition. In these patients, current treatment options are often limited by suboptimal topical efficacy or unnecessary systemic exposure. There has been a notable lack of innovation in the field of topical, rectal treatments designed to deliver active substances locally without systemic absorption; indeed, no new treatments have been approved in several years, especially in the USA [6].

These limitations highlight the need for novel therapeutic strategies, particularly those targeting local inflammatory pathways while minimizing systemic exposure.

Current therapeutic strategies for distal ulcerative colitis and ulcerative proctitis include locally acting corticosteroid formulations, among which rectal budesonide preparations represent an important treatment option due to their potent topical anti-inflammatory activity and reduced systemic exposure compared with conventional corticosteroids [7]. Budesonide is available in different rectal formulations, including foams and suppositories, which have demonstrated efficacy in inducing clinical and endoscopic remission in patients with mild-to-moderate distal disease [3,8]. However, despite their favourable safety profile, limitations such as incomplete mucosal coverage, variable retention time, and reduced efficacy in some patients highlight the need for novel topical approaches that provide sustained drug delivery and enhanced local activity [9]. In this context, the development of an in situ gelling formulation containing Rifamycin SV may represent an innovative strategy to improve local drug retention and therapeutic efficacy within the inflamed colonic mucosa.

Preclinical models play a crucial role in understanding disease mechanisms and evaluating new therapeutic approaches. Among these, the oxazolone-induced colitis model is widely used as it reproduces key features of human UC, including superficial mucosal inflammation, epithelial barrier disruption, and a predominant Th2-type immune response [10]. This model is therefore particularly suitable for investigating compounds targeting mucosal immune activation and epithelial injury [11].

Emerging evidence suggests that both microbial factors and host immune responses contribute to intestinal inflammation, and therapies combining antimicrobial and anti-inflammatory properties may offer additional benefits [12,13,14]. Rifamycin SV has demonstrated efficacy in intestinal inflammatory conditions due to its dual activity, including modulation of inflammatory pathways through activation of the pregnane X receptor (PXR) and the NF-kB pathway [15,16,17]. Notably, preclinical studies have shown that oral administration of rifamycin SV can significantly ameliorate the local signs of chemically induced colitis in rat models, further supporting its therapeutic potential [18]. These findings support the concept that locally acting agents with combined antimicrobial and immunomodulatory effects may represent a promising therapeutic strategy in inflammatory bowel diseases [19,20].

CB-01-35 is a novel formulation of rifamycin SV, which is liquid at room temperature and turns to gel at body temperature, with demonstrated in vitro antibacterial activity against microorganisms implicated in intestinal inflammation [15,18]. In addition, its potential anti-inflammatory properties suggest a dual mechanism of action that may be particularly relevant in conditions characterized by mucosal immune activation and barrier dysfunction [15,18].

Based on this evidence, the present study aimed to evaluate the therapeutic efficacy of intracolonic CB-01-35 administration in an acute oxazolone-induced colitis model in rats as a proof-of-concept. This approach has the potential to represent a first-in-class strategic advance in the topical treatment of distal ulcerative colitis and ulcerative proctitis, combining a dual mechanism of action (antibiotic and anti-inflammatory effect) with precision delivery and mucosal adherence, with the goal of improving both efficacy and tolerability, given the neutral pH of rifamycin enemas.

## 2. Materials and Methods

### 2.1. Compound Formulation

CB-01-35 enema and related Vehicle are identical to the CB-01-25 formulation and its Vehicle described in previous publications [18] and were provided by Cosmo S.p.A., Lainate (MI), Italy. The enema consisted of a liquid formulation characterized by in situ gelling properties. These formulations were prepared according to the patented technology with the combination of three polymers: the first polymer (Poloxamer 407) gels or creates a structured state after contact with the bio-relevant medium or at physiological temperature; its action is further strengthened by the interaction of the second polymer (Sodium alginate) with the target site, where the presence of specific trigger ions strengthens the interaction with the environmental medium of the other, resulting in an expansion of the polymer mixture’s capability of forming a gel or a structured state.

Upon administration and contact with body cavity, characterized by a physiological temperature (i.e., ≥34 °C), the third polymer (Sodium carboxymethyl cellulose) further ensures and strengthens the bio-adhesion, grafting the pharmaceutical composition to the body tissue [18].

This dual-triggered gelation and robust bio-adhesion establish a stable matrix at the application site, significantly prolonging residence time of active ingredients.

Moreover, the CB-01-35 enema has a neutral pH (about 7), which represents the optimal pH to ensure biocompatibility and decreased irritation effects. Conversely, all mesalamine enemas have a slightly acidic pH (about 4 to 5) due to inherent instability of the molecule at higher pHs; as a result, mesalamine enemas can sometimes cause discomfort and burning sensation to patients because of the effect of acidic medium on the inflamed and/or ulcerated mucosa.

Dexamethasone (Soldesam 4 mg/mL EuroGenerici, Ravenna, Italy) was acquired from a local pharmacy.

### 2.2. Animals

Seventeen female Wistar rats (body weight 190–200 g) were obtained from an accredited animal facility and housed in groups of 2–3 per cage under controlled environmental conditions (temperature 22 ± 2 °C, relative humidity 55 ± 10%, 12 h light/dark cycle). Animals had ad libitum access to standard laboratory chow and water throughout the experimental period. Health status was monitored daily, and all efforts were made to minimize stress and discomfort. Female Wistar rats were selected for this study due to their documented high susceptibility and reproducible response to oxazolone-induced colitis, with minimal mortality compared with murine models. Females were chosen exclusively to ensure body-weight homogeneity, thereby reducing inter-individual pharmacological variability during rectal dosing.

All experimental procedures were conducted in accordance with the European Directive 2010/63/EU on the protection of animals used for scientific purposes and complied with national regulations. The study protocol was reviewed and approved by the institutional animal care and use committee (IACUC), and all procedures adhered to internationally accepted guidelines for the care and use of laboratory animals.

Although the research protocol authorized by the Italian Ministry of Health (Permit number: 116/2023-PR) permitted a larger animal cohort, the sample size for this proof-of-concept study was intentionally reduced in accordance with the 3Rs principles. This decision was guided by preliminary unpublished in vivo gelation and pharmacokinetic data showing minimal inter-individual variability and highly consistent local tissue exposure, allowing clear preliminary biological signals to be evaluated prior to larger confirmatory trials.

### 2.3. Induction of Colitis

For the induction of colitis, rats were shaved on the dorsal area and pre-sensitized by topical application of 0.3 mL of an acetone–ethanol solution (1:4) containing 12 mg of oxazolone (Sigma-Aldrich, Milan, Italy) on day −7, followed by a second identical application on day −6. On day 0, colitis was induced by intracolonic (IC) administration of 250 μL of 50% ethanol containing 9 mg of oxazolone. Animals were sacrificed three days after induction (day 3) [21].

### 2.4. Treatments

Following induction of colitis (day 0), animals were randomly allocated to the different experimental groups before treatment initiation. Subsequently, animals were treated with a therapeutic regimen of test compounds, administered either intracolonically (IC) or intraperitoneally (IP) once daily from day 0 to day 2, for a total of 3 consecutive days. Treatment administration began on the same day as oxazolone instillation. Rats were euthanized on day 3. The administration of treatments was carried out by an investigator who was blinded to the experimental groups, in order to minimize potential bias during the study procedures.

Experimental groups included: (i) Intracolonic (IC) administration of CB-01-35 (2 mL/rat); (ii) IC administration of CB-01-35 vehicle (2 mL/rat); (iii) IC administration of a commercial formulation of mesalamine foam (Asacol foam, 2.5 mL/rat) as a positive control for the local delivery (2.5 mL/rat); and (iv) IP administration of dexamethasone dissolved in water (200 μL/rat) as a positive control group. An additional group of untreated animals (*n* = 2) that did not receive oxazolone was included as a healthy reference for all outcome measures.

Throughout the treatment period, body weight was recorded daily, and animals were monitored for clinical signs of disease and general health status.

### 2.5. Clinical Assessment

Clinical evaluations and monitoring of experimental parameters were performed by an operator blinded to the treatment allocation. Animals were weighed on day −7 and daily throughout the three-day treatment period. Body weight (BW) variation was calculated by normalizing each animal’s weight to its value on day 0, and the area under the curve (AUC) of daily BW changes was determined for each treatment group. During the same period, rats were monitored daily for clinical condition and stool consistency.

Stool scores were assigned daily for each animal on a scale from 0 (normal) to 2 (maximal severity), where 0 indicated well-formed pellets, 1 indicated loose stools, and 2 indicated liquid or bloody stools. A cumulative stool score was calculated by summing daily scores over the study duration. Clinical scores were also recorded daily on a scale from 0 (healthy) to 4 (severe disease), based on the following criteria: 0, no hunching or weight loss; 1, mild hunching or weight loss (1–5%); 2, moderate hunching or weight loss (6–10%); 3, moderate hunching, decreased mobility or weight loss (11–20%); 4, severe hunching, decreased mobility or weight loss (>20%). The AUCs of daily clinical scores were calculated for each treatment group.

The Disease Activity Index (DAI) was calculated daily for each animal as the sum of the clinical and stool scores, and the AUC of the DAI was subsequently determined for each treatment group.

### 2.6. Histological Analysis

The entire colon, from the caecum to the anus, was carefully isolated and cleared of mesentery, vessels, and adipose tissue. A 10 cm segment was measured and weighed after gently rinsing the lumen with saline.

For the assessment of mucosal damage, the distal colon was opened longitudinally with scissors and positioned with the mucosal surface facing upward. The mucosal damage area, corresponding to the necrotic area, was measured using a digital caliper and expressed in mm^2^. Macroscopic damage was also evaluated using a semi-quantitative macroscopic damage score (MDS) ranging from 0 to 6, according to the following criteria: 0, no damage; 1, local hyperaemia and/or edema; 2, linear ulcer involving less than half the width of the colon; 3, linear ulcer involving more than half the width of the colon; 4, circular ulcer < 1 cm; 5, circular ulcer between 1 and 2 cm; 6, circular ulcer > 2 cm.

Following macroscopic evaluation, the colon was weighed and fixed in 10% buffered formalin, as instructed by the pathologist. Formalin-fixed tissues were embedded in paraffin, and paraffin blocks were prepared. From each block, two to three transverse colon sections (10–12 μm thick) were obtained, mounted on slides, and stained with hematoxylin and eosin (H&E).

Histological evaluation and scoring were performed by an experienced pathologist who was completely unaware of the animal identification and corresponding treatment groups, according to the recommendations of the ARRIVE guidelines. Histological evaluation was performed using a semi-quantitative scoring system as follows: 0, no inflammation and no structural alterations; 1, mild submucosal leukocyte infiltration, mild edema, and submucosal erosion; 2, moderate leukocyte infiltration extending to the muscular layer, moderate edema, muscular layer ulceration, and mucosal thickening without ulceration; 3, severe transmural leukocyte infiltration, marked edema, and transmural ulceration.

For histological evaluation, three representative sections were analyzed for each animal. Within each section, two representative microscopic fields were examined according to the predefined histopathological scoring criteria.

### 2.7. Statistical Analysis

Statistical analyses were performed using GraphPad Prism version 9.0 for Windows (GraphPad Software, San Diego, CA, USA).

Given the streamlined sample size of this exploratory proof-of-concept study, formal statistical tests for normality were deemed underpowered. Consequently, to identify preliminary biological signals, statistical evaluations were performed using planned pairwise comparisons (two-tailed unpaired Student’s *t*-test) directly comparing active treatment groups against the Vehicle control. Data are presented as mean ± SD with 95% confidence intervals (95% CI). Statistical significance was defined as *p* < 0.05.

## 3. Results

### 3.1. Body Weight

**Figure 1:**
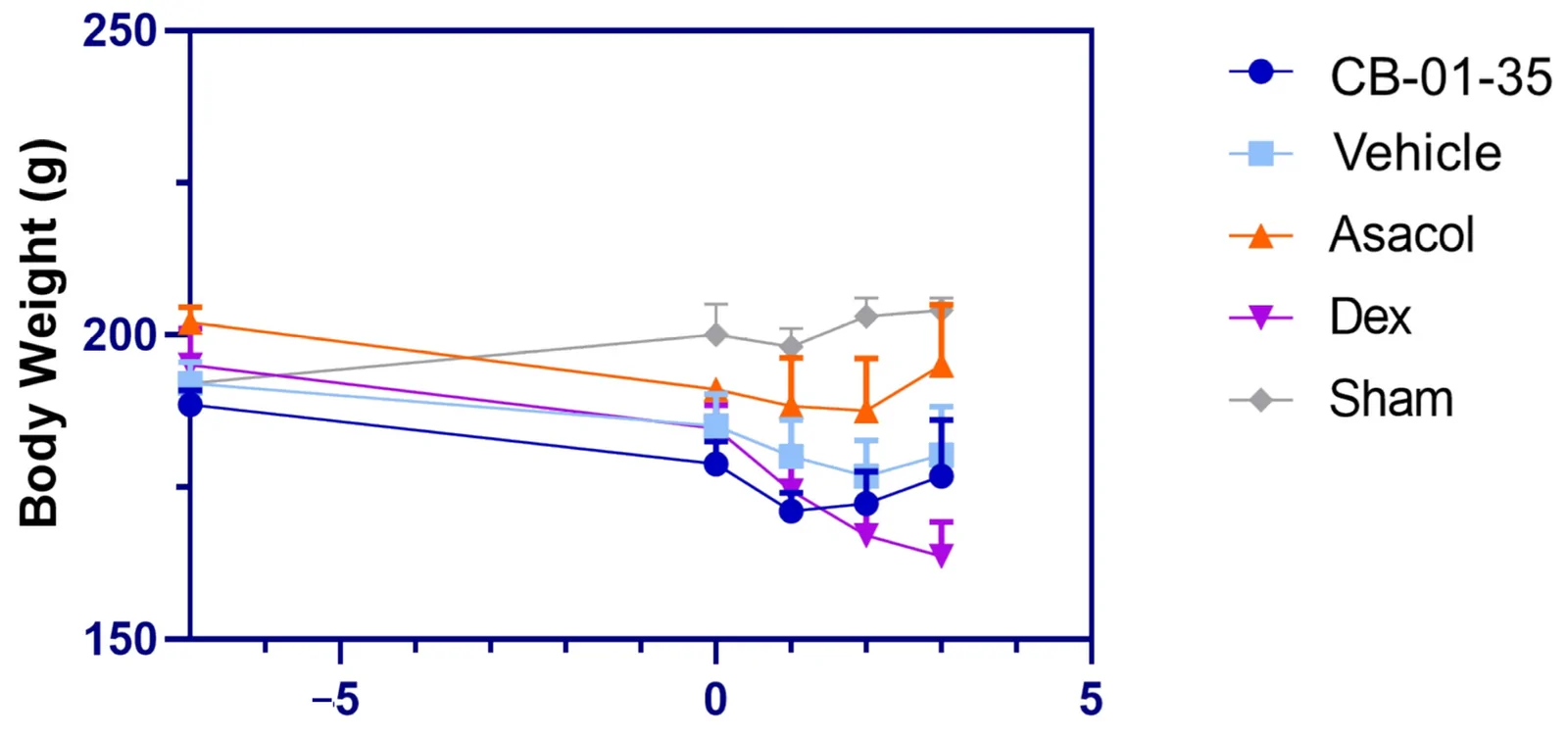
Effects of test compounds on BW variation over time in a rat model of oxazolone-induced colitis. Data are graphed as mean and SEM. Experimental groups included: CB-01-35-treated animals (*n* = 4), Vehicle group (*n* = 4), Asacol-treated animals (*n* = 4), Dex-treated animals (*n* = 3), and untreated sham controls (*n* = 2). For the evaluation of statistical significance, the unpaired T-test was used.

Body weight was monitored throughout the experimental period to assess general health status and treatment tolerability. All groups showed a similar pattern of body weight variation over time (Figure 1). Although a slight decrease in body weight was observed following disease induction (day 0), no significant differences were detected among the treatment groups (CB-01-35, Vehicle, Asacol, and dexamethasone) compared with the sham group. No treatment significantly impacted body weight compared with Vehicle, while dexamethasone showed a trend toward a greater reduction. These findings suggest that none of the treatments mitigated weight loss associated with colitis, suggesting a similar overall systemic impact across experimental conditions.

### 3.2. Colon Weight

**Figure 2:**
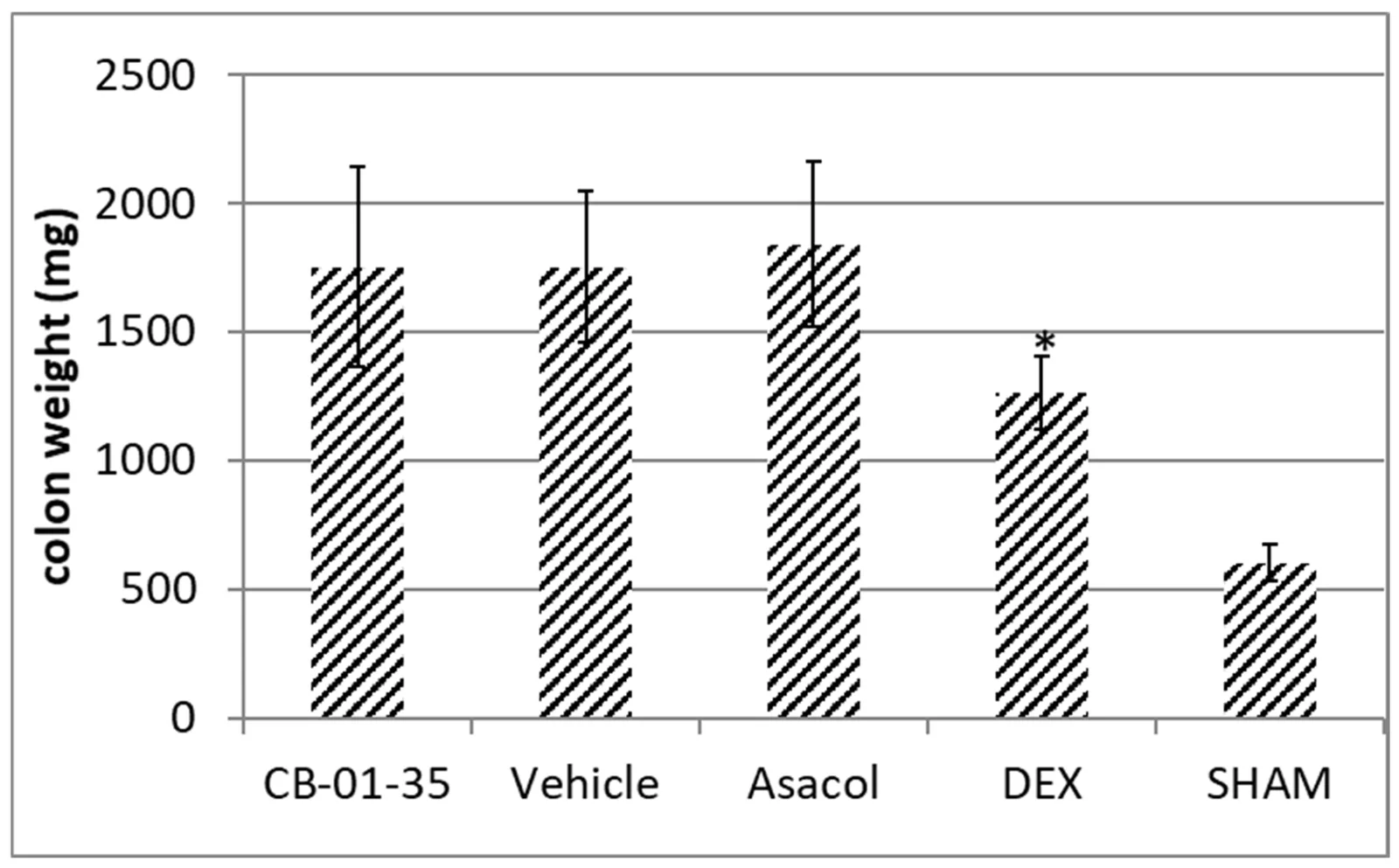
Effects of test compounds on colon weight in a rat model of oxazolone-induced colitis. Experimental groups included: CB-01-35-treated animals (*n* = 4), Vehicle group (*n* = 4), Asacol-treated animals (*n* = 4), Dex-treated animals (*n* = 3), and untreated sham controls (*n* = 2). For the evaluation of statistical significance, the unpaired T-test was used. Confidence Intervals of 95% (95% CI): Vehicle [1281, 2224], CB-01-35 [1134, 2369], Asacol [1042, 2638], Dexamethasone [908, 1615], Sham [−35, 1235]. * *p* < 0.05 vs. Vehicle.

Colon weight was evaluated as a marker of inflammation severity and was normalized to colon length to account for differences in intestinal size among experimental groups (Figure 2). No significant difference was observed between the CB-01-35-treated group and the Vehicle group (*p* = 0.998), indicating that intracolonic CB-01-35 did not improve this parameter. Similarly, treatment with Asacol did not reduce colon weight compared with Vehicle (*p* = 0.724). In contrast, dexamethasone treatment led to a significant decrease in colon weight compared with the Vehicle group (*p* = 0.048), consistent with its known potent anti-inflammatory effects. As expected, the sham group exhibited markedly lower colon weight than all colitis-induced groups.

### 3.3. Macroscopic Damage Score

**Figure 3:**
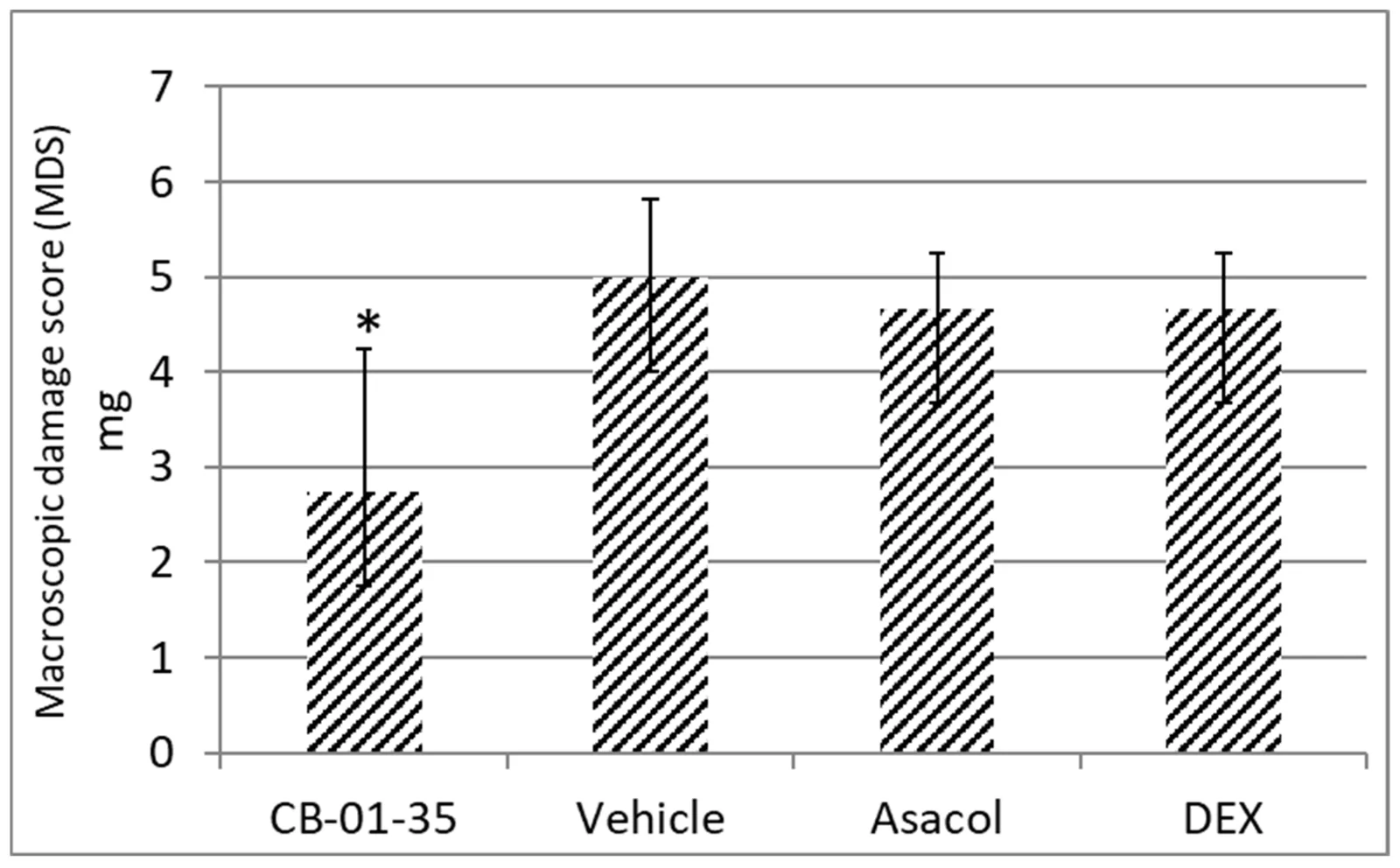
Effects of test compounds on Macroscopic Damage Score in a rat model of oxazolone-induced colitis. Experimental groups included: CB-01-35-treated animals (*n* = 4), Vehicle group (*n* = 4), Asacol-treated animals (*n* = 4), Dex-treated animals (*n* = 3). For the evaluation of statistical significance, the unpaired T-test was used. Confidence Intervals of 95% (95% CI): Vehicle [3.7, 6.3], CB-01-35 [0.36, 5.14], Asacol [3.2, 6.1], Dexamethasone [3.2, 6.1]. * *p* < 0.05 vs. Vehicle.

Macroscopic damage score (MDS) was assessed to evaluate the extent of colonic injury (Figure 3). Treatment with CB-01-35 administered IC resulted in a significant reduction in MDS compared with the Vehicle group (*p* = 0.039). Notably, CB-01-35 also demonstrated greater efficacy in reducing macroscopic damage when compared with both Asacol (*p* = 0.58) and dexamethasone (*p* = 0.58), which did not show any significant improvement relative to Vehicle. These results indicate that CB-01-35 effectively attenuates colonic macroscopic damage and demonstrates superior efficacy compared with the comparator treatments in this model.

### 3.4. Mucosal Damage Area

**Figure 4:**
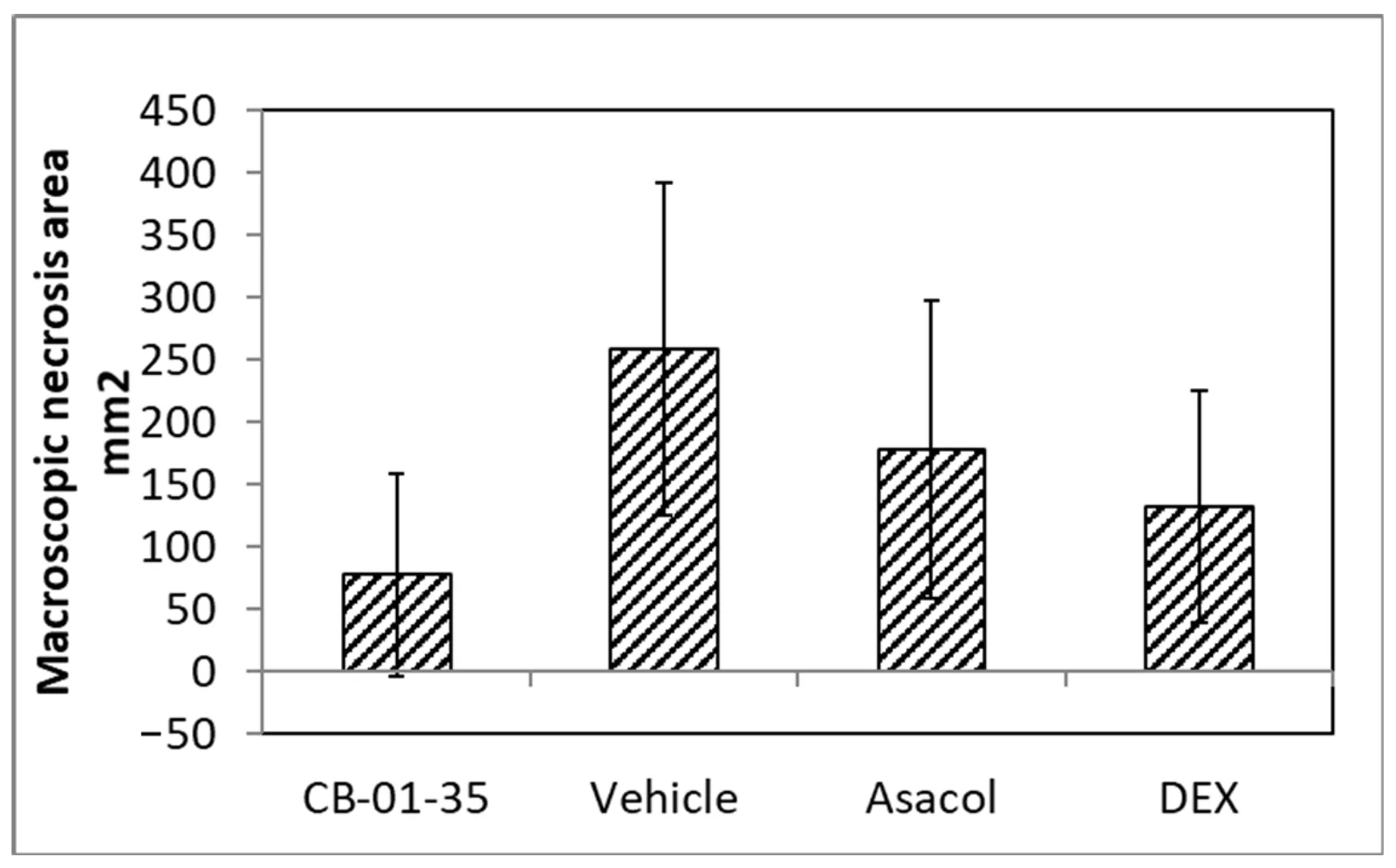
Effects of test compounds on mucosal damage area (Macroscopic Necrosis Area in mm^2^) in a rat model of oxazolone-induced colitis. Experimental groups included: CB-01-35-treated animals (*n* = 4), Vehicle group (*n* = 4), Asacol-treated animals (*n* = 4), Dex-treated animals (*n* = 3). For the evaluation of statistical significance, the unpaired T-test was used. Confidence Intervals of 95% (95% CI): Vehicle [46, 471], CB-01-35 [−52, 207], Asacol [−120, 476], Dexamethasone [−98, 362].

Mucosal damage area, expressed as necrotic area (mm^2^), was evaluated to assess the extent of colonic injury (Figure 4). Treatment with CB-01-35 administered IC reduced mucosal damage area levels compared with the Vehicle group, approaching statistical significance (*p* = 0.06). Although this trend suggests a potential protective effect, statistical significance was not reached, likely due to the limited number of animals per group. In contrast, neither Asacol (*p* = 0.449) nor dexamethasone (*p* = 0.224) treatments resulted in any improvement in macroscopic necrosis area compared with Vehicle.

### 3.5. Histological Score

**Figure 5:**
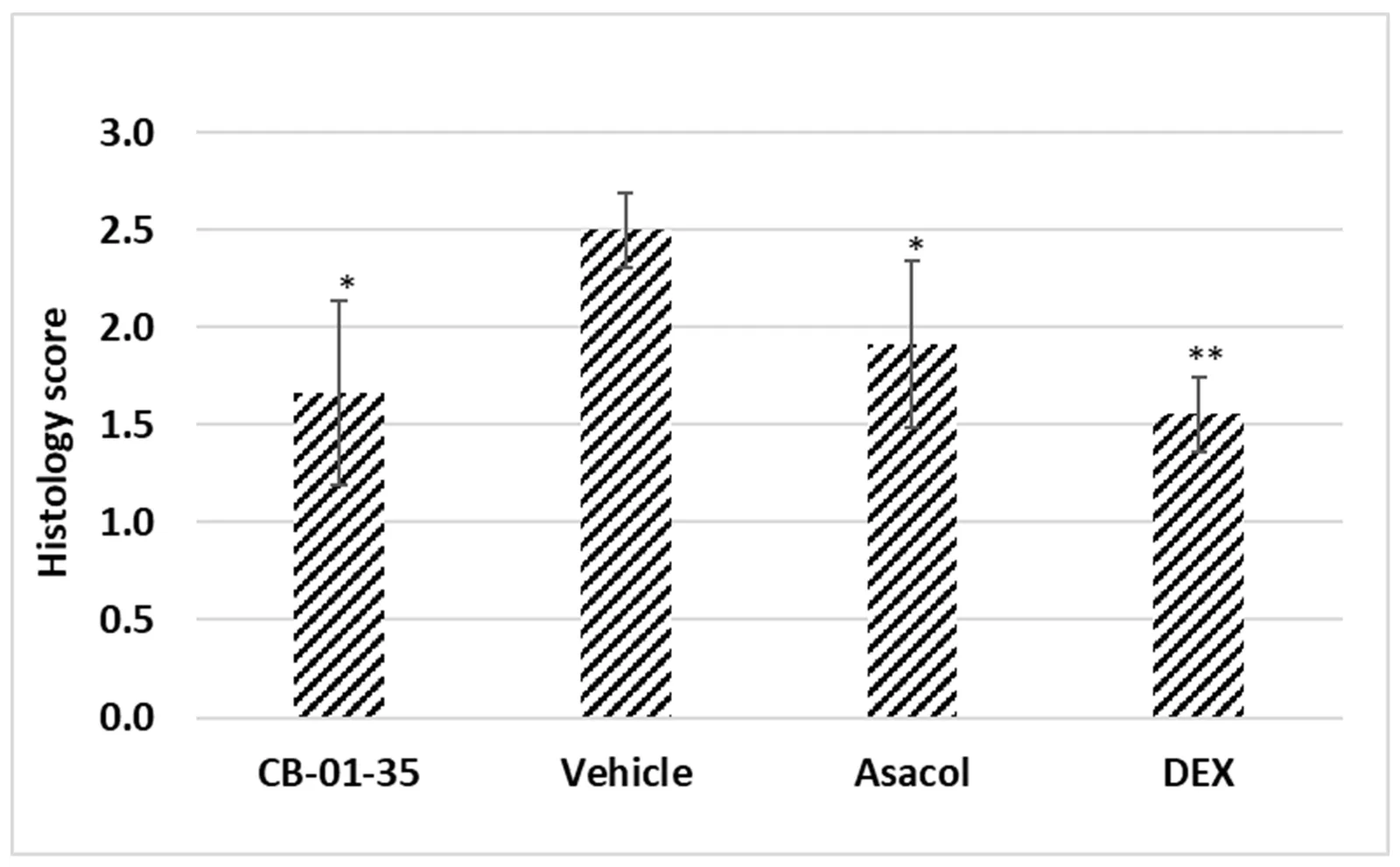
Effects of test compounds on histological score in a rat model of oxazolone-induced colitis. Experimental groups included: CB-01-35-treated animals (*n* = 4), Vehicle group (*n* = 4), Asacol-treated animals (*n* = 4), Dex-treated animals (*n* = 3), and untreated sham controls (*n* = 2). For the evaluation of statistical significance, the unpaired T-test was used. Confidence Intervals of 95% (95% CI): Vehicle [2.19, 2.81], CB-01-35 [0.92, 2.42], Asacol [1.15, 2.52], Dexamethasone [1.08, 2.03]; * *p* < 0.05; ** *p* < 0.01 vs. vehicle.

The beneficial effects observed with CB-01-35 on clinical parameters were confirmed by histological analysis of the colon (Figure 5). Hematoxylin and eosin (H&E)-stained sections, examined at 100× magnification, showed that rats treated with CB-01-35, as well as those treated with Asacol and dexamethasone, exhibited a significant reduction in histological score compared with the Vehicle group (*p* = 0.017, *p* = 0.03 and *p* = 0.001, respectively) (Figure 5).

**Figure 6:**
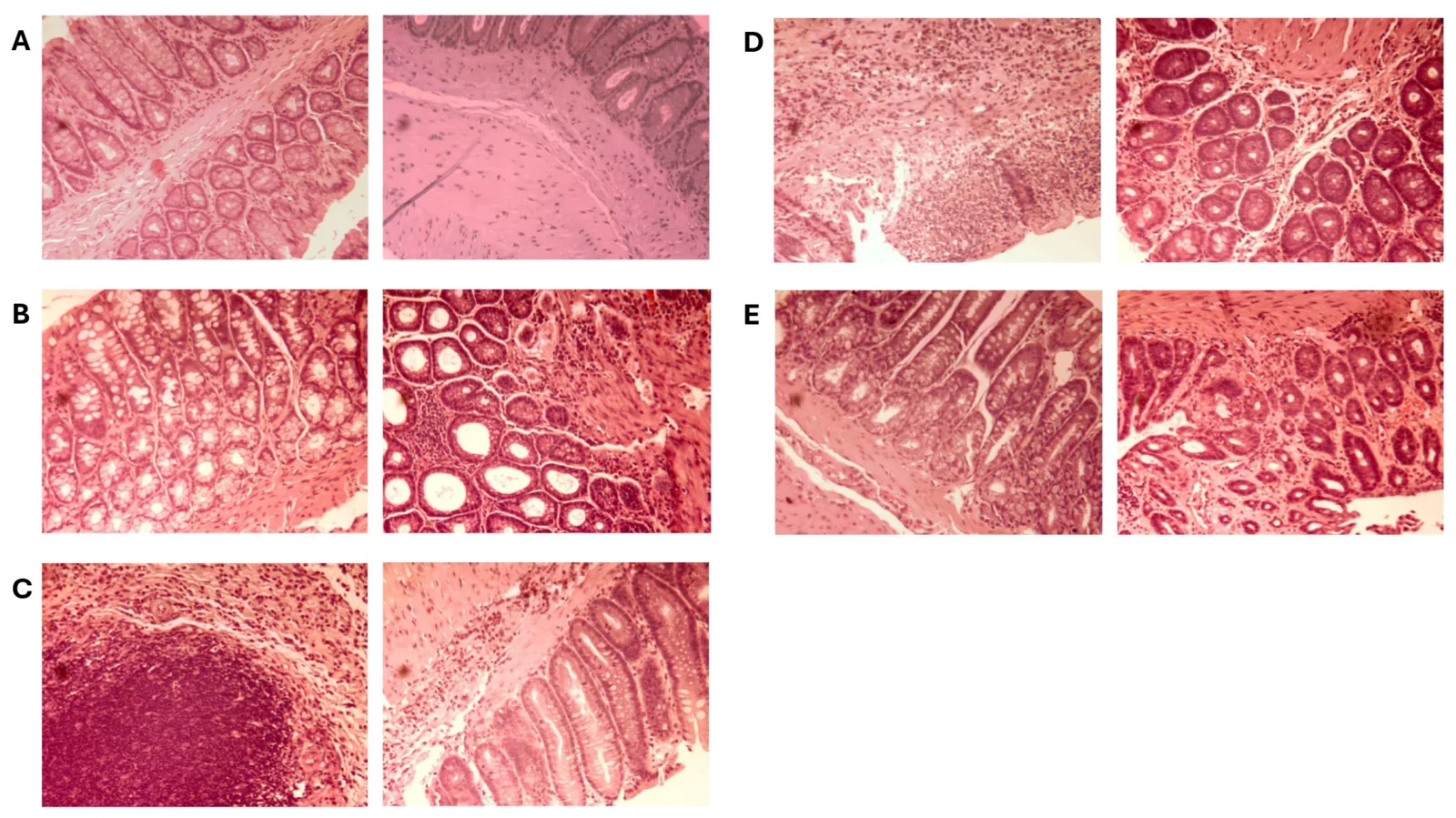
Representative images of H&E-stained colon sections (200× magnification) from: (**A**) healthy rats; (**B**) rats treated with CB-01-35; (**C**) Vehicle; (**D**) Asacol; and (**E**) Dexamethasone.

In particular, CB-01-35 treatment was associated with a marked decrease in leukocyte infiltration and tissue edema, as well as reduced epithelial erosion. These findings were supported by representative histological images (Figure 6B) which demonstrate improved tissue architecture and reduced inflammatory damage in treated groups compared with Vehicle (Figure 6C), confirming the protective effects observed in clinical disease activity measures.

## 4. Discussion

This study demonstrates that intracolonic administration of CB-01-35 reduces macroscopic and histological damage in an oxazolone-induced colitis model, supporting its potential as a locally acting therapeutic agent for distal colonic inflammation. The significant reduction in MDS, the positive trend in the reduction in the mucosal damage area, and the improvement in the histological score indicate a consistent anti-inflammatory effect at the tissue level.

The oxazolone model is characterized by a Th2-type immune response, which shares important features with human distal ulcerative colitis, including epithelial barrier disruption and mucosal inflammation [10]. In this context, the observed efficacy of CB-01-35 suggests that the compound may interfere with pathways involved in mucosal immune activation and epithelial injury. The use of the same in situ gelling formulation intended for clinical use in this model effectively mirrors the clinical objective of treating ulcerative proctitis and distal UC by delivering the active substance at the target site of action and keeping it in prolonged contact with the damaged mucosa.

A notable aspect of the results is the divergence between endpoints. While CB-01-35 significantly improved MDS and histological score, it did not significantly affect body weight or colon weight. This discrepancy may reflect the short duration of the study (three days), during which systemic or gross anatomical changes are less likely to manifest compared with localized mucosal healing. Furthermore, the lack of systemic impact aligns with the goal of developing a treatment that avoids systemic exposure, a key requirement for modern topical therapies in distal UC. The positive trend toward a reduction in mucosal damage area (*p* = 0.06) further supports a biological effect of CB-01-35. Although not reaching statistical significance, this finding is consistent with improvements observed in other endpoints and suggests that the lack of significance may be primarily driven by small group sizes rather than by the lack of efficacy.

Compared with reference treatments, CB-01-35 demonstrated beneficial effects on selected endpoints, with efficacy profiles comparable to those observed with the comparator treatments. Asacol, a standard topical therapy for distal ulcerative colitis, did not demonstrate clear improvements in macroscopic parameters in this model. These findings may reflect differences in pharmacokinetics or the specific inflammatory pathways activated in the oxazolone model. Furthermore, although mesalamine foam was evaluated as a clinical benchmark, it failed to achieve statistically significant mucosal healing and this discrepancy highlights the critical role of formulation mechanics in acute ulcerative conditions: commercial foams undergo rapid physiological clearance due to increased rectal motility and exudate, whereas our in situ gelling vehicle exhibits prolonged mucosal adhesion, providing sustained local drug exposure at the site of damage. Although no mechanistic investigations were performed in this study, the proposed mode of action for CB-01-35 involves a dual mechanism: an antibiotic effect combined with an anti-inflammatory response. In particular, the rapid mucosal healing observed is predominantly driven by direct, local anti-inflammatory activity, consistent with the known ability of rifamycin derivatives to activate the PXR and cross-inhibit NF-kappaB-mediated proinflammatory signaling pathways [17,22]. The improvement in histological score, particularly the reduction in leukocyte infiltration and epithelial damage, suggests a potential beneficial effect of CB-01-35 on local inflammatory processes in the colonic mucosa. This could involve inhibiting pro-inflammatory cytokine production (e.g., TNF-α, IL-6, IL-13) or interfering with immune cell recruitment and activation. This targeted delivery approach, mimicking the precision and mucosal adherence of a bio-adhesive gel, ensures that the drug remains at the target site of action. By delivering the active drug directly to the distal colon and rectum at a neutral pH, this strategy aims at optimizing both efficacy and tolerability. Nevertheless, while the consistent improvements observed in macroscopic and histological parameters support the preliminary proof-of-concept value of this investigation, the present study has some limitations that should be acknowledged. First, the relatively small sample size, particularly in the healthy control group, represents an important limitation that may have reduced the statistical power of the analyses and limited the ability to fully capture biological variability among animals. Therefore, the possibility of type I and type II statistical errors cannot be completely excluded, and the findings should be interpreted with caution. Larger cohorts with formal a priori power calculations will be used in subsequent confirmatory investigations.

A further limitation is the absence of molecular and biochemical analyses to investigate the mechanisms underlying the observed effects of CB-01-35. Although the macroscopic and histological improvements provide evidence of therapeutic activity in this experimental model, inflammatory mediators, immune-related pathways, and cellular responses were not directly assessed. Consequently, the potential anti-inflammatory mechanisms associated with CB-01-35 remain hypothetical and require further validation.

In addition, the impact of CB-01-35 treatment on the intestinal microbiota was not evaluated. Considering the antimicrobial activity of Rifamycin SV and the recognized contribution of gut microbial alterations to intestinal inflammation, the lack of microbiological characterization represents a limitation in the comprehensive understanding of the biological effects of this formulation.

Finally, the translational relevance of these findings should be considered within the limitations of the experimental model. The present study was performed in an acute oxazolone-induced rat model of colitis and was designed as a proof-of-concept investigation. Therefore, although CB-01-35 showed encouraging effects in this setting, these results cannot be directly extrapolated to clinical efficacy in patients with ulcerative proctitis. The complexity of human inflammatory bowel disease, including disease chronicity, interindividual variability, and differences in underlying pathophysiological mechanisms, requires caution when translating preclinical findings into the clinical setting.

These limitations should be considered in the interpretation of the present findings, which represent an initial proof-of-concept evaluation of CB-01-35 in an experimental model of colitis. Further studies incorporating larger experimental cohorts, molecular and inflammatory profiling, microbiome characterization, and validation in complementary preclinical models will be essential to confirm the robustness of these observations, elucidate the underlying mechanisms of action, and further support the translational potential of this rifamycin-based topical formulation.

In conclusion, CB-01-35 could represent a promising candidate for the local treatment of distal ulcerative colitis and ulcerative proctitis. These results provide a rationale for further investigation of this rifamycin-based topical therapy as a potential innovative approach to address the current need for improved local treatments in Inflammatory Bowel Disease.

## Data Availability

The original contributions presented in this study are included in the article. Further inquiries can be directed to the corresponding author.

## Abbreviations

The following abbreviations are used in this manuscript:

AUC	Area Under the Curve
BW	Body weight
DAI	Disease Activity Index
H&E	Hematoxylin and Eosin
IC	Intracolonically
IP	Intraperitoneally
MDS	Macroscopic Damage Score
PXR	Pregnane X Receptor
UC	Ulcerative Colitis
